# Research priorities in chronic lung allograft dysfunction: A machine learning-assisted bibliometric analysis

**DOI:** 10.1016/j.jhlto.2026.100645

**Published:** 2026-07-25

**Authors:** Kinan El Husseini, Margot Delin, Sixtine Decaux, Tiphaine Goletto, Lise Morer, Domitille Mouren, Mathilde Salpin, Gaëlle Weisenburger, Benjamin Coiffard, Claire Merveilleux du Vignaux, Jérôme Le Pavec, Benjamin Renaud-Picard, Antoine Roux, Adrien Tissot, Thomas Villeneuve, Arnaud Roussel, Pierre Mordant, Olivier Brugière, Hervé Mal, Vincent Bunel

**Affiliations:** a INSERM, UMR 1149, Centre de Recherche sur l′inflammation, Université Paris Cité, Paris, France; b Service de Pneumologie, et Transplantation Pulmonaire, AP-HP, Hôpital Bichat, Paris, France; c Department of Respiratory Medicine, and Lung Transplantation, APHM, Hôpital Nord, Université Aix-Marseille, Marseille, France; d Department of Pneumology, Hospices Civils de Lyon, Louis Pradel Hospital, Lyon, France; e Pulmonology and Lung Transplantation Department, Marie-Lannelongue Hospital, Groupe Hospitalier Paris-Saint-Joseph, Le Plessis-Robinson, France; f Paris-Sud University, School of Medicine, Paris-Saclay University, Le Kremlin Bicêtre, France; g INSERM, Research Unit UMR_S 1358, Paris-Sud University, Marie-Lannelongue Hospital, Groupe Hospitalier Paris-Saint-Joseph, Le Plessis Robinson, France; h Service de Pneumologie, CHU de Strasbourg, Strasbourg, France; i Service de Pneumologie, Foch Hospital, Suresnes, France; j Research Center Molecular Virology and Immunology, INRAE, UMR 0892, Paris, France; k Paris Translational Research Centre for Organ Transplantation, INSERM U970, PARCC, Université de Paris, Paris, France; l Nantes Université, CHU de Nantes, Institut du Thorax, Service de Pneumologie, Nantes, France; m INSERM 1064, Center for Research in Transplantation and Transitional Immunology, Nantes, France; n Department of Respiratory Medicine, University Hospital, Toulouse, France; o Toulouse Institute for Infectious and Inflammatory Diseases (Infinity), INSERM U1291, CNRS U5282, University of Toulouse, Toulouse, France; p Service de Chirurgie Vasculaire et Thoracique, AP-HP, Hôpital Bichat, Paris, France

**Keywords:** Chronic lung allograft dysfunction, Lung transplantation, Bibliometrics, Phenotype, Bronchiolitis obliterans syndrome

## Abstract

**Background:**

Chronic lung allograft dysfunction (CLAD), the leading cause of death beyond year one post-transplant, encompasses bronchiolitis obliterans syndrome (BOS), restrictive allograft syndrome (RAS), and mixed allograft syndrome (MAS), formalized by the 2019 ISHLT consensus. How thematic priorities have evolved and whether research effort tracks phenotype burden have not been systematically examined.

**Methods:**

From 1,851 CLAD-related papers (Web of Science, 1990-2025) assembled after AI-assisted relevance screening (validated against blinded expert adjudication), machine learning-based topic modeling using a biomedical language model identified thematic clusters; temporal trajectories were classified by statistical trend analysis; and a phenotype representation index (RI = corpus proportion ÷ clinical prevalence) was computed against 2 reference cohorts with bootstrap confidence intervals.

**Results:**

Twenty-four thematic clusters were identified (13 declining, 8 stable, 3 rising, 1 emerging). Rising topics included DSA/antibody-mediated rejection (119 papers), restrictive phenotype classification (126 papers), and translational CLAD pathogenesis (143 papers), reflecting momentum predating the 2019 consensus. Donor-derived cell-free DNA was the sole emerging topic (21 papers). Among 950 phenotype-focused papers, BOS was proportionally represented (RI 1.13 vs Leuven; 1.05 vs Toronto). RAS representation was denominator-dependent (RI 0.75-0.99). MAS was substantially under-represented in both cohorts (RI 0.33 vs Leuven; 0.41 vs Toronto).

**Conclusions:**

Transformer-based topic modeling reveals a directional shift toward immunological mechanisms and phenotype-specific research that classical keyword approaches cannot resolve. Research effort is broadly proportionate to clinical burden for BOS but substantially below for MAS, a gap possibly attributable to pathophysiological differences, diagnostic complexity, limited case volume, and recency of phenotype formalization that multicenter studies may address.

## Background

Lung transplantation remains the only definitive therapy for a broad spectrum of end-stage pulmonary diseases, yet long-term outcomes lag behind other solid organ transplant programs. Chronic lung allograft dysfunction (CLAD), the umbrella designation for progressive, non-infectious allograft injury, is the principal barrier to improving long-term survival. Affecting approximately 50% of recipients by 5 years post-transplant, CLAD accounts for the majority of deaths beyond the first year.^1^ No pharmacological therapy has demonstrated durable efficacy in reversing or halting established disease, and median survival following CLAD diagnosis remains poor.^2^

The clinical conceptualization of CLAD has evolved substantially over 3 decades. The bronchiolitis obliterans syndrome (BOS) working formulation, anchored to a sustained decline in forced expiratory volume in 1 second (FEV₁), was established in 1993^3^ and its staging criteria revised in 2002^4^ and 2007.^5^ Restrictive allograft syndrome (RAS), defined by peripheral or pleuroparenchymal fibrosis with a restrictive spirometric pattern, was first formally described in 2011.^6^ A third, mixed phenotype (MAS) combining obstructive and restrictive physiology was subsequently characterized.^7^ In 2019, the ISHLT consensus formalized this 3-phenotype framework, BOS, RAS, and MAS, recognizing each as clinically, physiologically, and prognostically distinct, with RAS and MAS conferring substantially shorter survival than BOS.^2^, ^8^

Despite this conceptual advance, whether the 2019 ISHLT framework has reshaped the thematic priorities of the CLAD literature, stimulating investigation of newly recognized phenotypes, redirecting mechanistic inquiry, or primarily consolidating existing clinical classification, has not been characterized systematically. One specific and quantifiable aspect of this broader question is whether published research effort tracks the clinical prevalence of each phenotype and whether any disproportion has persisted or narrowed since formal phenotype recognition in 2019.

Bibliometric analysis of large literature corpora has previously been applied to map research trajectories in transplantation and similar fields.^9^ Classical co-citation and keyword co-occurrence methods identify structural relationships between papers but cannot resolve semantic proximity where shared concepts are expressed in heterogeneous vocabulary. Transformer-based topic modeling, specifically BERTopic, which derives topic clusters from dense abstract embeddings generated by a domain-adapted biomedical language model,^10^ addresses this limitation directly. Complementing this approach, large language model (LLM)-assisted classification applied to the full corpus enables systematic, context-aware categorization beyond what keyword search alone permits.

We therefore conducted a systematic bibliometric study of the CLAD literature, addressing 3 research questions: (1) how have the publication volume, geographic distribution, and journal landscape of CLAD research evolved from 1990 to 2025; (2) how is the literature structured thematically and temporally, in particular, does the 2019 ISHLT consensus correspond to a directional shift in research priorities; and (3) is research effort proportionate to the clinical prevalence of each CLAD phenotype, and how has this distribution evolved since formal phenotype recognition in 2019?

## Materials and methods

### Search strategy and data source

The Web of Science (WOS) Core Collection was searched on March 13th, 2026, for publications containing CLAD-related terminology in the title, abstract, or keywords, without language restriction, covering the period January 1990 to December 2025. The complete search string is provided in Supplementary Materials. Records were exported in plain text format and processed in R. The full screening flow is reported according to PRISMA 2020 guidelines.^11^

### Relevance screening

A 3-stage cascade was applied to the 5,062 retrieved records. Stage 0 (rule-based): records containing hematopoietic stem cell transplantation-specific terminology were excluded (*n* = 50; 1 additional record excluded on manual review), retaining 5,011. Stage 1 (term-based): records lacking any recognized CLAD-related term in the title or abstract were excluded (*n* = 2,451), retaining 2,561. Stage 2 (LLM-based): LLM-assisted title and abstract screening has been validated for systematic review workflows^12^; each abstract was submitted to Claude Sonnet (claude-sonnet-4-6; Anthropic, San Francisco, CA) via the Anthropic Batch API with a structured classification prompt; papers classified as RELEVANT were retained (*n* = 1,851), and those classified as PERIPHERAL (*n* = 710) were excluded. The final corpus comprises 1,851 papers spanning 1990 to 2025.

Stages 0 and 1 were deterministic rule-based filters and used no language model; language-model classification was confined to Stage 2. To verify the Stage 2 classifications, a stratified random sample of 200 records (100 classified RELEVANT, 100 classified PERIPHERAL) was independently adjudicated by one of the authors (KEH), a transplant pulmonologist blinded to the model's decision and its justification. Agreement was near-perfect (Cohen's κ = 0.97). None of the 100 records the model had excluded as PERIPHERAL were reclassified as relevant (false-exclusion rate 0%, 95% CI 0%-3.7%), and 97% of records classified RELEVANT were confirmed (positive predictive value 97%, 95% CI 91.5%-99.0%); these figures correspond to an estimated screening sensitivity of 100% and specificity of 93%.

### Bibliometric analysis

Standard bibliometric indicators—annual publication counts, country-level output, journal distribution, citation analysis, and international collaboration networks—were computed using the bibliometrix R package.^13^ Country-level collaboration networks were constructed for countries contributing ≥ 10 publications, with edges representing country pairs sharing ≥ 3 co-authored papers; the Fruchterman–Reingold algorithm was used for network layout. The 20 most-cited papers were identified from cited reference data extracted from WOS records. Author affiliation addresses were additionally resolved to continent and city. Each paper was attributed to every continent and city represented among its affiliations.

### Topic modeling

Topic modeling was performed using BERTopic^10^ with domain-specific PubMedBERT sentence embeddings.^14^ PubMedBERT is a biomedical language model used solely to convert abstract text into numerical embeddings and does not entail any search of the PubMed/MEDLINE database. The leaf cluster-selection method was chosen a priori as more appropriate for a specialty corpus in which semantically adjacent subdomains carry distinct clinical meaning; full preprocessing pipeline, technical parameters, and validation metrics are provided in Supplementary Methods.

### Research-category classification

Each paper was assigned a document type from its WOS classification: original article, review, proceedings paper, and editorial/letter. Each thematic topic was also mapped onto one of four predefined research domains: basic/translational, diagnostics/monitoring, therapeutics, or clinical/outcomes. Within-year proportions of each document type and research domain were computed across 1995 to 2025 to describe compositional change.

### Dynamic topic analysis

Temporal topic proportions were computed using BERTopic’s dynamic topic modeling framework with annual time slices derived from each paper’s publication year. Mann-Kendall trend tests^15^, ^16^ were applied to each topic’s proportional time series (significance threshold α = 0.10). Four trajectory classes were defined a priori: *rising* (significant positive trend, Kendall’s τ > 0); *declining* (significant negative trend, τ < 0); *stable* (no significant trend); and *emerging* (series length < 10 years with positive trend direction, insufficient series length for Mann-Kendall significance testing). A fifth class, *phasic* (peak in the middle third of the series, ≥ 10% relative decline in the final third, minimum series length 12 years), was pre-specified but not observed in this corpus. Because proportional rather than absolute frequency was used, a declining trajectory reflects decreasing share of a growing corpus, not necessarily fewer papers per year.

### Phenotype representation index

Each corpus paper was classified by primary CLAD phenotype focus using structured keyword matching applied to concatenated titles and abstracts. Curated term lists were defined for each phenotype: BOS terms included “bronchiolitis obliterans syndrome,” “bos,” and related obstructive descriptors; RAS terms included “restrictive allograft syndrome,” “ras,” “pleuroparenchymal fibroelastosis,” and “ppfe” MAS terms included “mixed allograft syndrome,” “mixed clad,” and cognate expressions. Papers matching terms from 2 or more phenotypes were classified as *comparative*; those matching no phenotype-specific term were classified as *none*.

The representation index (RI) for each phenotype was computed as its corpus proportion, restricted to single-phenotype papers (BOS + RAS + MAS; *n* = 950), divided by its clinical prevalence in 2 independent reference cohorts: Leuven (Verleden et al, *n* = 268 CLAD patients)^17^ and Toronto (Levy et al, *n* = 174 CLAD patients).^18^ Comparative and none papers were excluded from the RI denominator; clinical prevalence denominators were normalized to phenotype-classified patients only. Bootstrap 95% confidence intervals were computed with 10,000 resamples. An RI of 1.0 denotes research effort proportionate to clinical burden; RI < 1 indicates under-representation.

## Results

### Corpus characteristics and publication trends

**Figure 1:**
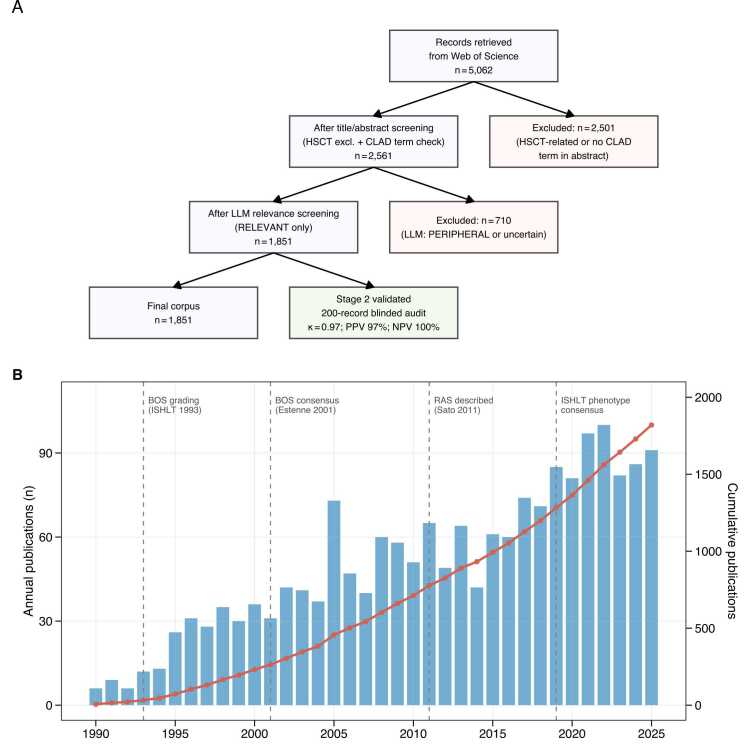
*Corpus overview.***(A)** PRISMA flow diagram of the 3-stage relevance screening pipeline. **(B)** Annual publication volume (bars, left axis) and cumulative output (line, right axis) for the CLAD literature identified in Web of Science, 1990-2025 (*n* = 1,851 papers). Key clinical milestones are indicated by dashed vertical lines. *Abbreviations:* BOS, bronchiolitis obliterans syndrome; CLAD, chronic lung allograft dysfunction; HSCT, haematopoietic stem cell transplantation; ISHLT, International Society for Heart and Lung Transplantation; LLM, large language model; RAS, restrictive allograft syndrome; WOS, Web of Science.

The final corpus comprised 1,851 papers published between 1990 and 2025 (Fig. 1). Annual output grew in 2 broad phases: a first expansion from approximately 15 papers per year in 1990 to a peak of approximately 70 per year in 2005 to 2006, followed by a plateau averaging approximately 60 papers per year through 2010 to 2018. After the 2019 ISHLT consensus, mean annual output increased to approximately 88 papers per year, a 48% rise relative to the preceding decade; growth remained roughly linear without a discrete inflection point. The 2 most-cited papers in the corpus were the 2007 ISHLT revision of the nomenclature of the diagnosis of lung rejection (Stewart et al, 954 citations)^5^ and the original 1993 BOS working formulation (Cooper et al, 658 citations) (Table 1).^3^

**Table 1:** Top 20 Most-Cited Papers in the CLAD Literature

Rank	First author	Year	Journal	Title	Cited (WoS)	Topic
1	Stewart, S	2007	J Heart Lung Transpl	Revision of the 1996 working formulation for the standardization of nomenclature in the diagnosis of lung rejection	954	Surveillance bronchoscopy and transbronchial biopsy
2	Cooper, JD	1993	J Heart Lung Transpl	A working formulation for the standardization of nomenclature and for clinical staging of chronic dysfunction in lung allografts	658	BOS staging and FEV1 decline
3	Meyer, KC	2014	Eur Respir J	An international ISHLT/ATS/ERS clinical practice guideline: diagnosis and management of bronchiolitis obliterans syndrome	425	Obliterative bronchiolitis—pathophysiology and overview
4	Verleden, GM	2014	J Heart Lung Transpl	A new classification system for chronic lung allograft dysfunction	424	Obliterative bronchiolitis—pathophysiology and overview
5	Burke, CM	1984	Chest	Post-transplant obliterative bronchiolitis and other late lung sequelae in human heart-lung transplantation	401	Retransplantation and long-term survival
6	Sato, M	2011	J Heart Lung Transpl	Restrictive allograft syndrome (RAS): A novel form of chronic lung allograft dysfunction	362	RAS/restrictive phenotype and CLAD classification
7	Bando, K	1995	J Thorac Cardiovasc Sur	Obliterative bronchiolitis after lung and heart-lung transplantation – an analysis of risk factors and management	338	Obliterative bronchiolitis—pathophysiology and overview
8	Burlingham, WJ	2007	J Clin Invest	IL-17-dependent cellular immunity to collagen type V predisposes to obliterative bronchiolitis in human lung transplants	336	Peripheral blood immune profiling in BOS
9	Daud, SA	2007	Am J Resp Crit Care	Impact of immediate primary lung allograft dysfunction on bronchiolitis obliterans syndrome	304	BOS staging and FEV1 decline
10	Khalifah, AP	2004	Am J Resp Crit Care	Respiratory viral infections are a distinct risk for bronchiolitis obliterans syndrome and death	260	Obliterative bronchiolitis—pathophysiology and overview
11	Davis, RD	2003	J Thorac Cardiovasc Sur	Improved lung allograft function after fundoplication in patients with gastroesophageal reflux disease undergoing lung transplantation	258	Gastroesophageal reflux and aspiration
12	Charlson, ES	2012	Am J Resp Crit Care	Lung-enriched Organisms and Aberrant Bacterial and Fungal Respiratory Microbiota after Lung Transplant	255	Airway microbiome and bacterial colonization
13	Sharples, LD	2002	J Heart Lung Transpl	Risk factors for bronchiolitis obliterans: A systematic review of recent publications	254	Obliterative bronchiolitis—pathophysiology and overview
14	Cantu, E III	2004	Ann Thorac Surg	Early Fundoplication prevents chronic allograft dysfunction in patients with gastroesophageal reflux disease	254	Gastroesophageal reflux and aspiration
15	D'Ovidio, F	2005	J Thorac Cardiovasc Sur	Bile acid aspiration and the development of bronchiolitis obliterans after lung transplantation	249	Gastroesophageal reflux and aspiration
16	Gerhardt, SG	2003	Am J Resp Crit Care	Maintenance azithromycin therapy for bronchiolitis obliterans syndrome - Results of a pilot study	246	Azithromycin treatment
17	Verleden, GM	2006	Am J Resp Crit Care	Azithromycin reduces airway neutrophilia and interleukin-8 in patients with bronchiolitis obliterans syndrome	245	Azithromycin treatment
18	Barker, AF	2014	New Engl J Med	Obliterative Bronchiolitis	233	Obliterative bronchiolitis—pathophysiology and overview
19	Girgis, RE	1996	J Heart Lung Transpl	Risk factors for the development of obliterative bronchiolitis after lung transplantation	231	BOS staging and FEV1 decline
20	Heng, D	1998	J Heart Lung Transpl	Bronchiolitis obliterans syndrome: Incidence, natural history, prognosis, and risk factors	229	BOS staging and FEV1 decline

†Cited (WoS): total citation count in Web of Science as of the search date. Topic: BERTopic cluster assignment; proceedings papers assigned post-hoc by nearest-centroid cosine similarity.

**Figure 2:**
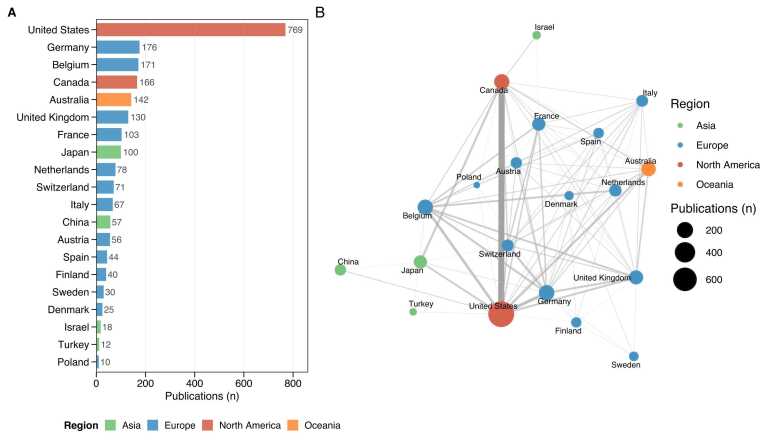
*Geographic distribution and international collaboration.***(A)** Top 20 countries by number of publications. Bars are colored by world region. **(B)** International collaboration network among countries with ≥ 10 publications. Node size is proportional to publication count; edge width is proportional to the number of co-authored papers. Only country pairs with ≥ 3 co-authorships are shown. Layout generated by the Fruchterman-Reingold algorithm.

The United States contributed the greatest publication volume and occupied a central position in the international collaboration network (Fig. 2). At the continental level, North America (50.7% of papers) and Europe (42.8%) dominated the literature, with smaller contributions from Asia (11.5%) and Oceania (7.9%) and minimal representation from South America and Africa (Supplementary Table 3). The highest-output cities were Leuven (141 papers), St Louis (139), Toronto (131), Durham (100), and Los Angeles (80), reflecting a small group of high-volume specialist centers that includes both cohorts used in the phenotype representation analysis (Leuven and Toronto).

The *Journal of Heart and Lung Transplantation* accounted for the largest share of corpus output (314 papers; 17.0%), followed by *Transplantation* (168; 9.1%) and the *American Journal of Transplantation* (149; 8.1%); the full journal distribution is presented in Supplementary Table 1.

### Thematic structure of the CLAD literature

**Figure 3:**
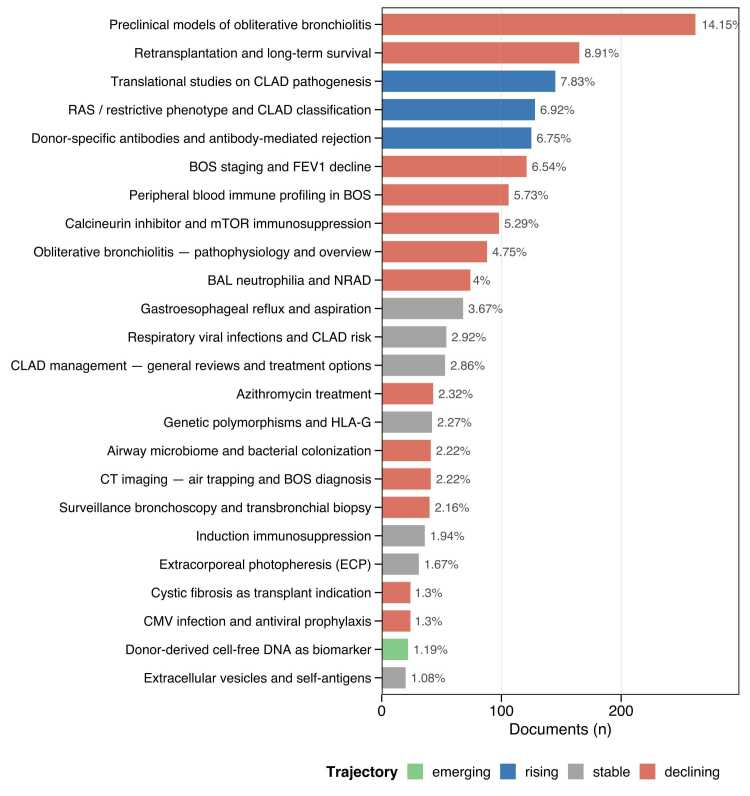
Thematic structure of the CLAD literature. Horizontal bar chart of the 24 BERTopic thematic clusters, ranked by document count. Bar color indicates the Mann-Kendall temporal trajectory of each topic (see legend). Percentage labels indicate each topic’s share of the full corpus (*n* = 1,851).

BERTopic identified 24 thematic clusters across the 1,714 natural language processing-eligible documents (Fig. 3). Trajectory classification yielded 13 declining, 8 stable, 3 rising, and 1 emerging topic; no phasic topics were observed (Table 2). The largest single cluster was preclinical obliterative bronchiolitis (OB) models (262 papers; 14.15% of corpus), followed by retransplantation and long-term survival (165 papers; 8.91%), translational CLAD pathogenesis (145 papers; 7.83%), and RAS/restrictive phenotype classification (128 papers; 6.92%).

**Table 2:** Summary of Thematic Clusters Identified in the CLAD Literature

Label	Trajectory	*n*	% corpus	Peak year	Kendall τ	Top 5 terms
Preclinical models of obliterative bronchiolitis	Declining	262	14.15%	2003	−0.54	ob; cell; airway; mouse; epithelial
Retransplantation and long-term survival	Declining	165	8.91%	2014	−0.48	survival; retransplantation; donor; outcome; age
Translational studies on CLAD pathogenesis	Rising	145	7.83%	2020	0.74	clad; cell; bal; chronic lung allograft dysfunction; associate
RAS / restrictive phenotype and CLAD classification	Rising	128	6.92%	2020	0.35	clad; ras; phenotype; ct; chronic lung allograft dysfunction
Donor-specific antibodies and antibody-mediated rejection	Rising	125	6.75%	2022	0.42	dsa; antibody; amr; hla; antihla
BOS staging and FEV₁ decline	Declining	121	6.54%	2003	−0.69	bos; fev1; risk; decline; onset
Peripheral blood immune profiling in BOS	Declining	106	5.73%	2019	−0.33	cell; bos; blood; stable; increase
Calcineurin inhibitor and mTOR immunosuppression	Declining	98	5.29%	2003	−0.29	tacrolimus; cyclosporine; everolimus; therapy; trial
Obliterative bronchiolitis — pathophysiology and overview	Declining	88	4.75%	2003	−0.51	bos; chronic; bronchiolitis obliterans; airway; syndrome
BAL neutrophilia and NRAD	Declining	74	4%	2003	−0.49	bos; bal; neutrophil; level; fluid
Gastroesophageal reflux and aspiration	Stable	68	3.67%	2009	−0.23	reflux; aspiration; fundoplication; gastric; gastroesophageal reflux
Respiratory viral infections and CLAD risk	Stable	54	2.92%	2020	−0.10	infection; virus; respiratory; rsv; viral
CLAD management — general reviews and treatment options	Stable	53	2.86%	2019	0.20	clad; review; survival; chronic lung allograft dysfunction; treatment
Azithromycin treatment	Declining	43	2.32%	2010	−0.37	azithromycin; azi; fev1; treatment; bos
Genetic polymorphisms and HLA-G	Stable	42	2.27%	2024	0.16	polymorphism; hlag; ltx; snps; associate
CT imaging — air trapping and BOS diagnosis	Declining	41	2.22%	2003	−0.57	ct; trapping; air; scan; bos
Airway microbiome and bacterial colonization	Declining	41	2.22%	2012	−0.54	microbiome; colonization; cf; pseudomonas; bacterial
Surveillance bronchoscopy and transbronchial biopsy	Declining	40	2.16%	2003	−0.83	biopsy; rejection; grade; transbronchial; surveillance
Induction immunosuppression	Stable	36	1.94%	2014	−0.02	alemtuzumab; induction; atg; therapy; receive
Extracorporeal photopheresis (ECP)	Stable	31	1.67%	2023	0.13	ecp; treatment; extracorporeal photopheresis; decline; therapy
CMV infection and antiviral prophylaxis	Declining	24	1.3%	2003	−0.69	cmv; prophylaxis; ganciclovir; cytomegalovirus; pneumonitis
Cystic fibrosis as transplant indication	Declining	24	1.3%	2021	−0.73	cystic; cf; fibrosis; survival; bronchiectasis
Donor-derived cell-free DNA as biomarker	Emerging	22	1.19%	2021	0.24	ddcfdna; injury; dna; clad; plasma
Extracellular vesicles and self-antigens	Stable	20	1.08%	2019	−0.07	exosome; sag; ltxrs; selfantigen; circulate

† *n*: number of papers assigned to the topic. % corpus: proportion of the full corpus (*n* 1,851).

‡ Trajectory classified by Mann-Kendall trend test (α = 0.10) on annual proportional frequency. Kendall τ: Kendall’s tau statistic from the Mann-Kendall test (positive = upward trend). NA for topics with series length < 4 years. Emerging: positive trend direction (τ > 0) but series length < 10 years, insufficient for Mann–Kendall significance testing. Phasic: peak in middle third of series with ≥10% relative decline in final third (minimum 12 years of data required). Top 5 terms: highest-ranking c-TF-IDF tokens for the topic. Four topics were pre-specified as sentinel areas to validate BERTopic recovery (DSA/antibody-mediated rejection; BOS staging and FEV₁ decline; gastro-esophageal reflux and aspiration; azithromycin treatment); all 4 were recovered.

Original articles accounted for 83.9% of the corpus and reviews for 9.1%, with conference proceedings (6.3%) representing a declining share over time and reviews an increasing share in the most recent decade (Supplementary Figure 1A). Grouped into research domains (Supplementary Table 2), basic and translational research formed the largest share (30.1% of papers), followed by diagnostics and monitoring (27.2%), clinical and outcomes research (26.5%), and therapeutics (16.2%). Therapeutics remained a smaller fraction throughout (Supplementary Figure 1B).

*Trajectory definitions:* Emerging, positive trend direction (Kendall’s τ > 0) but series length < 10 years, insufficient for Mann-Kendall significance testing; Rising, statistically significant positive trend (τ > 0, *p* < 0.10); Stable, no significant directional trend (*p* ≥ 0.10); Declining, statistically significant negative trend (τ < 0, *p* < 0.10). A fifth class (Phasic: peak in middle third, ≥ 10% relative decline in final third, minimum 12 years required) was pre-specified but not observed in this corpus. Proportional (not absolute) publication frequency was used for trajectory classification; a declining trajectory, therefore, reflects decreasing share of the growing corpus, not necessarily fewer papers per year.

*Abbreviations:* ATG, anti-thymocyte globulin; BAL, bronchoalveolar lavage; BOS, bronchiolitis obliterans syndrome; CLAD, chronic lung allograft dysfunction; CMV, cytomegalovirus; CT, computed tomography; DSA, donor-specific antibodies; ECP, extracorporeal photopheresis; EMT, epithelial-to-mesenchymal transition; FEV₁, forced expiratory volume in 1 second; GERD, gastro-oesophageal reflux disease; HLA, human leukocyte antigen; MAS, mixed allograft syndrome; NRAD, neutrophilic reversible allograft dysfunction; OB, obliterative bronchiolitis; RAS, restrictive allograft syndrome.

### Temporal dynamics

**Figure 4:**
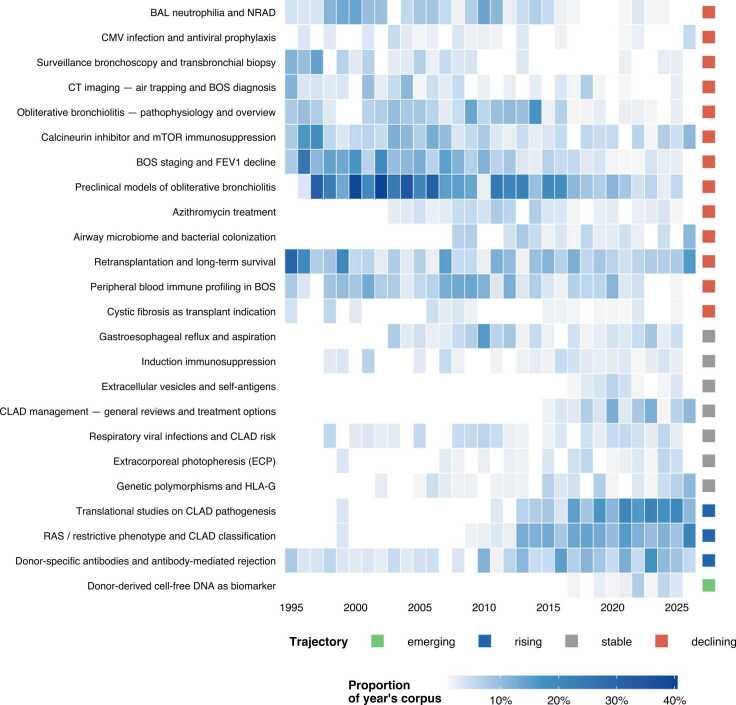
*Temporal dynamics of CLAD research topics.* Heatmap of proportional topic representation over time. Each cell shows the fraction of that year’s corpus assigned to the corresponding topic. Topics are sorted by trajectory class (top to bottom: emerging, rising, stable, declining) and by peak year within each class. The right-hand column of colored squares indicates trajectory category. *Abbreviations:* See Fig. 3.

The 3 rising topics all reached peak proportional representation between 2020 and 2022 (Fig. 4). Translational studies on CLAD pathogenesis, encompassing epithelial-to-mesenchymal transition and fibrogenesis (143 papers), and RAS/restrictive phenotype classification (126 papers) both peaked in 2020. DSA/antibody-mediated rejection (119 papers) showed the most recent peak, in 2022. The single emerging topic, donor-derived cell-free DNA as a biomarker (21 papers; 7-year series, onset approximately 2015), had too short a series for Mann-Kendall significance testing but maintained a positive trend direction throughout its observation window, with peak proportional representation in 2021.

Among the 13 declining topics, 2 of the largest clusters peaked in 2003: preclinical OB models (262 papers) and BOS staging/FEV₁ decline (121 papers); same for calcineurin inhibitor and mTOR regimens (98 papers). Retransplantation and long-term survival (165 papers) peaked in 2014, while the azithromycin treatment cluster (43 papers) peaked in 2010. Because proportional frequency informed trajectory classification, declining trajectory reflects a decreasing share of a growing corpus; absolute annual output in these topics may have remained stable or declined more slowly than their proportional representation implies.

### Phenotype representation

Of the 1,851 corpus papers, 950 (51.3%) contained keyword evidence of a single CLAD phenotype: BOS 806 (84.8% of the single-phenotype subgroup), RAS 117 (12.3%), and MAS 27 (2.8%). A further 228 papers (12.3%) matched terms from 2 or more phenotypes (*comparative*), and 673 (36.4%) contained no phenotype-specific term (*none*). Representation indices were computed among the 950 single-phenotype papers (Table 3).

**Table 3:** Phenotype Representation Index for CLAD Research Topics

Phenotype	Denominator	*n* (tagged)	Corpus %	95% CI	Clinical prev.	RI	95% CI	Interpretation
BOS/Obstructive	Leuven (*n* = 268)	806	84.8%	82.5%-87.1%	75%	1.13	1.1-1.16	Over-represented
RAS/Restrictive	Leuven (*n* = 268)	117	12.3%	10.2%-14.4%	16.4%	0.75	0.62-0.88	Under-represented
MAS/Mixed	Leuven (*n* = 268)	27	2.8%	1.8%-3.9%	8.7%	0.33	0.21-0.45	Under-represented
BOS/Obstructive	Toronto (*n* = 174)	806	84.8%	82.5%-87.1%	80.6%	1.05	1.02-1.08	Over-represented
RAS/Restrictive	Toronto (*n* = 174)	117	12.3%	10.2%-14.4%	12.4%	0.99	0.82-1.16	At parity
MAS/Mixed	Toronto (*n* = 174)	27	2.8%	1.8%-3.9%	7%	0.41	0.26-0.56	Under-represented

† *n* (tagged): papers assigned to each phenotype among phenotype-tagged papers only (*n* = 950 of 1,851). Proportions computed within this tagged subset.

‡ Clinical prevalence (norm.): proportion of phenotype-classified CLAD patients in the reference cohort, normalized to exclude undefined/unclassified cases. RI, Representation Index = corpus proportion/clinical prevalence. RI = 1.0 indicates research effort proportionate to clinical burden. 95% CI from 10,000 bootstrap resamples. Leuven: Verleden SE et al Clin Transplant. 2020;34:e13781. Toronto: Levy L et al J Heart Lung Transplant. 2020;39(8):761–770.

BOS was proportionally represented relative to its clinical burden in both reference cohorts (RI 1.13, 95% CI 1.10-1.16 vs Leuven^17^; RI 1.05, 95% CI 1.02-1.08 vs Toronto^18^). RAS representation was denominator-dependent: modestly under-represented against the Leuven cohort, in which RAS prevalence was higher (RI 0.75, 95% CI 0.62-0.88), but at near parity against Toronto (RI 0.99, 95% CI 0.82-1.16). MAS was substantially under-represented in both cohorts (RI 0.33, 95% CI 0.21-0.45 vs Leuven; RI 0.41, 95% CI 0.26-0.56 vs Toronto). The magnitude of MAS under-representation was consistent across both reference cohorts and robust across the full range of bootstrap resamples.

## Discussion

The CLAD literature grew steadily through the study period, with a moderate step-up in annual output after 2019. The more telling signal is thematic. The 3 rising trajectories, DSA/antibody-mediated rejection, restrictive phenotype classification, and translational studies, reflect research programs that were already gathering momentum before the 2019 ISHLT consensus formalized the 3-phenotype framework^2^: RAS was first described in 2011^6^ and the immunological basis of CLAD injury had been under active investigation since the mid-2000s.^19^ The consensus most plausibly codified what the literature was already moving toward rather than initiating new directions. That the post-2019 period shows continued growth in these same trajectories is consistent with formalized phenotypic definitions making previously heterogeneous clinical entities more tractable research targets, though the bibliometric record alone cannot establish the direction of that effect.

The rising DSA/AMR trajectory reflects a broader reconceptualization of CLAD pathogenesis. Historically framed as primarily a structural airway remodeling process driven by alloimmune injury,^20^ CLAD is increasingly understood to involve antibody-mediated mechanisms tractable to donor-recipient compatibility optimization, monitoring of circulating donor-specific antibodies, and potentially B-cell-directed therapies.^21^, ^22^, ^23^ The rise of this cluster in parallel with the restrictive phenotype classification cluster suggests that immunological reconceptualization and phenotype reclassification have proceeded as a coupled development, both driven by recognition that the BOS-only framework was insufficient.

The treatment literature is correspondingly sparse. Combining the azithromycin, extracorporeal photopheresis, and general CLAD management clusters accounts for approximately 6.8% of the corpus, a proportion that sits in stark contrast to the disease's clinical weight. This gap is not unique to the bibliometric record: Cravedi et al recently documented that clinical trial activity targeting acute rejection has outpaced CLAD-directed trials by a factor of 6 since 2017 and attributed this in part to poorly characterized pathogenic mechanisms and the difficulty of early phenotypic diagnosis.^24^ Both factors are visible in the present data. A field that has only recently stabilized its phenotypic taxonomy and that systematically underrepresents its most diagnostically complex phenotype in the mechanistic literature is not yet positioned to generate the clinical trial infrastructure that CLAD's prognosis demands. Phenotype-stratified mechanistic research is therefore not an end but a prerequisite for therapeutic development. The research-domain composition corroborates this imbalance: therapeutics accounted for the smallest share of the corpus (16.2%) and showed no proportional growth across the study period.

The MAS under-representation finding (RI 0.33-0.41 in both cohorts), in a phenotype with documented prognostic severity,^8^, ^25^ is the sharpest quantitative result of this study and warrants careful interpretation. Three non-exclusive explanations are plausible. First, diagnostic complexity: MAS requires CT evidence of pleuroparenchymal fibrosis superimposed on physiological obstruction,^2^ a combination that demands systematic phenotyping across serial imaging and spirometry that is inconsistently available in retrospective series. Second, case volume: with MAS comprising less than 10% of CLAD cases at specialist centers,^17^, ^18^ most programs lack the statistical power for single-center mechanistic studies; this creates a structural impediment to publication that is independent of scientific interest. Third, temporal lag: MAS was not formally defined until after 2019, so most of the observation window predates the phenotype’s existence as a codified research target. That said, the post-2019 period, during which MAS could be a prospectively defined study endpoint, does not yet show a compensatory rise in the MAS-focused literature, suggesting the gap reflects more than lag alone. Multicentre registries with prospective phenotype classification, harmonized imaging protocols, and sufficient case volume to power phenotype-stratified analyses represent the most direct path to closing this gap.

The 2 largest declining clusters, preclinical OB models and BOS staging/FEV₁ spirometry criteria, both peaked in 2003. For FEV₁-based BOS staging, the proportional decline most plausibly reflects successful translation into clinical infrastructure: once grading criteria were formalized and widely adopted, the investigative frontier shifted from staging refinement toward phenotype differentiation, mechanism, and treatment; precisely the topics now constituting the rising trajectories. For preclinical OB models, a different dynamic is at play. The classic heterotopic tracheal transplant model^26^ generated foundational mechanistic insights but incompletely recapitulates the clinical CLAD syndrome, and the field’s increasing translational orientation has diversified preclinical approaches rather than converged on a single platform. Orthotopic murine single-lung transplantation more faithfully recapitulates the clinical syndrome but imposes considerable microsurgical demands that restrict its use to specialist laboratories and limit reproducibility across research teams^27^; even within this model, bronchiolitis obliterans lesions develop in a minority of allografts, and survival endpoints are not feasible, constraining experimental throughput.^28^ The proportional decline in this cluster, therefore, most likely reflects these challenges as well as corpus dilution, clinical, immunological, and phenotyping research expanding the denominator, rather than saturation of research questions.

More ambiguous is the declining trajectory of the retransplantation and long-term survival cluster (peak 2014): this may reflect an emerging clinical consensus on retransplant candidacy criteria,^25^ or it may represent a gap that will re-emerge as phenotype-specific long-term outcome data accumulate. Crucially, the declining trajectory here reflects the decreasing proportional share of a growing corpus; absolute annual output in these topics may be stable rather than falling.

Several methodological considerations limit interpretation of our results. This analysis drew on a single database (WOS), which may under-represent non-English literature, conference proceedings, gray literature, and studies indexed in PubMed/MEDLINE but not in WOS. Stage 2 relevance screening was performed by a LLM; although blinded adjudication showed near-perfect agreement, residual misclassification of individual records cannot be entirely excluded. The phenotype representation index is bounded by the inherent limitation of keyword classification, which cannot formally distinguish a phenotype term appearing as the paper’s primary focus from its incidental use in background or eligibility criteria, and by the stability of the clinical prevalence denominators: both reference cohorts are single-center populations with specific case mixes (bilateral lung transplant-predominant, European and North American), and the MAS under-representation finding in particular rests on comparison with only these 2 published phenotype cohorts; phenotype prevalence estimates are known to vary across programs. Phenotype distribution is notably sensitive to center-specific induction and maintenance immunosuppression strategies and to differing surveillance and diagnostic practices. The RI therefore quantifies disproportion relative to these specific cohorts and should not be interpreted as a universal benchmark. Finally, the emerging cfDNA topic (*n* = 21, 7-year series) is too small for trajectory inference beyond directional observation, and its clinical trajectory will be determined by ongoing prospective biomarker studies^29^ rather than by bibliometric signals at this time scale.

More broadly, transformer-based topic modeling resolves semantic heterogeneity that keyword co-occurrence cannot but does not eliminate it entirely; cluster assignments reflect patterns in abstract language and may not fully capture the conceptual boundaries that domain experts would draw. Topic labels represent our interpretation of the top c-TF-IDF terms and representative documents for each cluster, and trajectory findings should therefore be understood as directional signals in the bibliometric record rather than definitive claims about the field's underlying intellectual structure.

The findings suggest several tractable research priorities. The MAS and RAS gaps point to a need for multicenter mechanistic studies, phenotype-stratified biomarker development, and clinical trial designs built around the 2019 3-phenotype framework rather than the legacy BOS-only paradigm. The geographic distribution of the literature reveals a field concentrated in a small number of high-volume specialist centers in Europe and North America, together accounting for most publications, with substantially lower output from Asia-Pacific programs and emerging transplant centers globally. These high-volume programs share characteristics that may not be representative of transplant practice or phenotype distributions worldwide. International data harmonization, including standardized prospective phenotyping and imaging protocols across programs of varying volume and case mix, would enable research priority setting grounded in the global needs of the transplant community.

## Financial support

This study received no dedicated funding.

## CRediT authorship contribution statement

Conceptualization, Data Curation, Formal Analysis, Methodology, Visualization, Writing – original draft: KEH. Validation, Writing – review & editing: MD, SD, TG, LM, DM, MS, GW, BC, XD, CMdV, JLP, BRP, ARoux, AT, TV, ARous, PM, OB, HM, VB.

## Declaration of Generative AI and AI-Assisted Technologies in the Writing Process

Generative AI was used to assist in developing the analysis pipeline (code), in Stage 2 relevance screening (as described in Methods and validated against blinded expert adjudication), and for language editing and drafting assistance during manuscript preparation. All AI-assisted outputs were reviewed and verified by the authors, who take full responsibility for the content.

## Conflicts of Interest statement

The authors report no conflict of interest relating to this study.

## Footnotes

Appendix A Supplementary data associated with this article can be found in the online version at doi:10.1016/j.jhlto.2026.100645.
