# Supplementary material for: Research priorities in chronic lung allograft dysfunction: A machine learning-assisted bibliometric analysis
Source: JHLT Open. 2026 Jul 25;14:100645. doi: 10.1016/j.jhlto.2026.100645 (PMC13476576; doi:10.1016/j.jhlto.2026.100645)
Supplement: Supplementary file 1 [file mmc1.docx]

# Supplementary Materials for: Research Priorities in Chronic Lung Allograft Dysfunction: A Machine Learning-Assisted Bibliometric Analysis

## Supplementary Methods

### Search strategy and data source

The Web of Science (WOS) Core Collection was searched on 13 March 2026 using the following topic-field (TS) search string, without language restriction, covering January 1990 to December 2025:

*TS = ("chronic lung allograft dysfunction" OR "CLAD" OR "bronchiolitis obliterans syndrome" OR "bronchiolitis obliterans" OR "obliterative bronchiolitis" OR "restrictive allograft syndrome" OR "mixed allograft syndrome") AND TS = ("lung transplant*" OR "lung allograft*" OR "pulmonary transplant*")*

Records were exported in plain text format. No language filter was applied at the search stage; non-English records were retained through all screening stages and included in the final corpus if they met relevance criteria. The full screening flow is reported according to PRISMA 2020 guidelines.^1^

### NLP preprocessing

Of the 1,851 corpus papers, 1,714 contained sufficient abstract text for natural language processing (NLP) analysis; 137 conference proceedings with absent or truncated abstracts were excluded from topic modelling training but retained for all other analyses. Abstracts were processed with scispaCy (en_core_sci_lg model)^2^ with multi-word biomedical expressions joined as single tokens (e.g., “bronchiolitis obliterans” → bronchiolitisobliterans). A dual-input design was applied: raw abstracts were used for semantic embedding, and preprocessed token sequences for class-based TF-IDF (c-TF-IDF) term extraction, separating representation learning from topic keyword summarisation.

### Topic modelling: technical parameters and validation

Abstracts were encoded with a PubMedBERT sentence encoder.^3^ Dimensionality was reduced with UMAP (n_neighbors = 15, n_components = 5, cosine metric)^4^ and clusters were identified with HDBSCAN (min_cluster_size = 15, cluster selection method: leaf).^5^ The leaf method, which yields finer-grained clusters than the default excess-of-mass approach, was selected a priori as more appropriate for a specialty corpus in which semantically adjacent subdomains carry distinct clinical meaning. Representative terms per topic were identified by c-TF-IDF applied to preprocessed token sequences.

Stability was assessed across 20 random seeds; mean pairwise adjusted Rand index (ARI) was 0.793 (SD 0.048; normalised mutual information 0.856), exceeding the pre-specified stability gate (ARI ≥ 0.70). Coherence was compared against a latent Dirichlet allocation (LDA) baseline at the optimal number of topics (k = 15): BERTopic achieved a higher Cᵥ coherence score (0.639 vs. 0.529). External validity was assessed by sentinel topic recovery: four topics were nominated before modelling (DSA/antibody-mediated rejection; BOS staging and FEV₁ decline; gastro-oesophageal reflux and aspiration; azithromycin treatment), and all four were recovered in the final solution. The 137 documents excluded from BERTopic training (primarily conference proceedings) were assigned to topics post-hoc by nearest-centroid cosine similarity against the trained topic embeddings.

### Phenotype classification: keyword term lists

Each paper was classified by primary CLAD phenotype focus using structured keyword matching applied to concatenated titles and abstracts. Curated term lists were defined for each phenotype:

- **BOS terms**: “bronchiolitis obliterans syndrome”, “bos” (word-boundary match), “obstructive clad”, “obstructive allograft”, “fev1 decline”, “bronchiolitis obliterans” (in lung transplant context).
- **RAS terms**: “restrictive allograft syndrome”, “ras” (word-boundary match), “pleuroparenchymal fibroelastosis”, “ppfe”, “restrictive clad”, “restrictive allograft”.
- **MAS terms**: “mixed allograft syndrome”, “mixed clad”, “mas” (word-boundary match), “mixed phenotype” (in CLAD context).

Papers matching terms from two or more phenotypes were classified as comparative; those matching no phenotype-specific term were classified as none. When a single phenotype was matched, a priority hierarchy (MAS > RAS > BOS) resolved assignment to prevent lower-specificity BOS terms from masking RAS or MAS classification.

### References

1.	Page MJ, McKenzie JE, Bossuyt PM, et al. The PRISMA 2020 statement: an updated guideline for reporting systematic reviews. *BMJ*. 2021;372:n71. doi:10.1136/bmj.n71

2.	Neumann M, King D, Beltagy I, Ammar W. ScispaCy: Fast and Robust Models for Biomedical Natural Language Processing. In: Demner-Fushman D, Cohen KB, Ananiadou S, Tsujii J, eds. *Proceedings of the 18th BioNLP Workshop and Shared Task*. Association for Computational Linguistics; 2019:319-327. doi:10.18653/v1/W19-5034

3.	Gu Y, Tinn R, Cheng H, et al. Domain-Specific Language Model Pretraining for Biomedical Natural Language Processing. *ACM Trans Comput Healthcare*. 2022;3(1):1-23. doi:10.1145/3458754

4.	McInnes L, Healy J, Melville J. UMAP: Uniform Manifold Approximation and Projection for Dimension Reduction. *arXiv*. Preprint posted online September 18, 2020:arXiv:1802.03426. doi:10.48550/arXiv.1802.03426

5.	McInnes L, Healy J, Astels S. hdbscan: Hierarchical density based clustering. *Journal of Open Source Software*. 2017;2(11):205. doi:10.21105/joss.00205

## Supplementary Results

### Supplementary Figures

**Supplementary Figure 1. Research-category composition of the CLAD literature over time.** Proportional stacked-area charts of the within-year composition of the corpus, 1995–2025; values are 3-year moving averages and each vertical axis shows the category's share of that year's publications. (A) By document type, from the Web of Science classification: original article, review, proceedings paper, and editorial/letter. (B) By research domain: each BERTopic thematic topic was assigned to one of four domains (basic/translational, diagnostics/monitoring, therapeutics, or clinical/outcomes; the full topic-to-domain mapping is given in Supplementary Table S2). Years before 1995 are omitted because of sparse annual output. *Abbreviations: BERTopic, neural topic modelling with class-based TF-IDF; CLAD, chronic lung allograft dysfunction.*

### Supplementary Tables

**Supplementary Table 1. Distribution of publications across journals (Web of Science corpus, n 1,851).**

| **Journal** | **Abbreviation** | **n** | **% corpus** |
| --- | --- | --- | --- |
| JOURNAL OF HEART AND LUNG TRANSPLANTATION | J HEART LUNG TRANSPL | 314 | 17% |
| TRANSPLANTATION | TRANSPLANTATION | 168 | 9.1% |
| AMERICAN JOURNAL OF TRANSPLANTATION | AM J TRANSPLANT | 149 | 8% |
| AMERICAN JOURNAL OF RESPIRATORY AND CRITICAL CARE MEDICINE | AM J RESP CRIT CARE | 76 | 4.1% |
| CLINICAL TRANSPLANTATION | CLIN TRANSPLANT | 53 | 2.9% |
| TRANSPLANTATION PROCEEDINGS | TRANSPL P/TRANSPLANT P | 51 | 2.8% |
| ANNALS OF THORACIC SURGERY | ANN THORAC SURG | 48 | 2.6% |
| JOURNAL OF THORACIC AND CARDIOVASCULAR SURGERY | J THORAC CARDIOV SUR | 47 | 2.5% |
| EUROPEAN RESPIRATORY JOURNAL | EUR RESPIR J | 42 | 2.3% |
| CHEST | CHEST | 40 | 2.2% |
| TRANSPLANT INTERNATIONAL | TRANSPL INT | 39 | 2.1% |
| TRANSPLANT IMMUNOLOGY | TRANSPL IMMUNOL | 36 | 1.9% |
| EUROPEAN JOURNAL OF CARDIO-THORACIC SURGERY | EUR J CARDIO-THORAC | 27 | 1.5% |
| CURRENT OPINION IN ORGAN TRANSPLANTATION | CURR OPIN ORGAN TRAN | 25 | 1.4% |
| PLOS ONE | PLOS ONE | 22 | 1.2% |
| FRONTIERS IN IMMUNOLOGY | FRONT IMMUNOL | 21 | 1.1% |
| TRANSPLANTATION DIRECT | TRANSPLANT DIRECT | 17 | 0.9% |
| JOURNAL OF IMMUNOLOGY | J IMMUNOL | 14 | 0.8% |
| HUMAN IMMUNOLOGY | HUM IMMUNOL | 13 | 0.7% |
| JOURNAL OF CLINICAL INVESTIGATION | J CLIN INVEST | 13 | 0.7% |
| RESPIRATORY MEDICINE | RESP MED | 13 | 0.7% |
| RESPIRATORY RESEARCH | RESP RES/RESPIR RES | 13 | 0.7% |
| SEMINARS IN RESPIRATORY AND CRITICAL CARE MEDICINE | SEMIN RESP CRIT CARE/ SEM RESP CRIT CARE M | 13 | 0.7% |
| JCI INSIGHT | JCI INSIGHT | 12 | 0.6% |
| JOURNAL OF THORACIC DISEASE | J THORAC DIS | 12 | 0.6% |
| THORAX | THORAX | 12 | 0.6% |
| TRANSPLANT INFECTIOUS DISEASE | TRANSPL INFECT DIS | 12 | 0.6% |
| AMERICAN JOURNAL OF PATHOLOGY | AM J PATHOL | 11 | 0.6% |
| FRONTIERS IN TRANSPLANTATION | FRONT TRANSPLANT | 10 | 0.5% |
| JOURNAL OF CLINICAL MEDICINE | J CLIN MED | 10 | 0.5% |
| RADIOLOGY | RADIOLOGY | 10 | 0.5% |
| AMERICAN JOURNAL OF RESPIRATORY CELL AND MOLECULAR BIOLOGY | AM J RESP CELL MOL | 9 | 0.5% |
| CLINICAL AND EXPERIMENTAL IMMUNOLOGY | CLIN EXP IMMUNOL | 9 | 0.5% |
| INTERNATIONAL IMMUNOPHARMACOLOGY | INT IMMUNOPHARMACOL | 9 | 0.5% |
| SCIENTIFIC REPORTS | SCI REP-UK | 9 | 0.5% |
| INTERACTIVE CARDIOVASCULAR AND THORACIC SURGERY | INTERACT CARDIOV TH | 8 | 0.4% |
| LUNG | LUNG | 8 | 0.4% |
| PEDIATRIC TRANSPLANTATION | PEDIATR TRANSPLANT | 8 | 0.4% |
| SURGERY TODAY | SURG TODAY | 8 | 0.4% |
| ERJ OPEN RESEARCH | ERJ OPEN RES | 7 | 0.4% |
| EXPERIMENTAL LUNG RESEARCH | EXP LUNG RES | 7 | 0.4% |
| INTERNATIONAL JOURNAL OF MOLECULAR SCIENCES | INT J MOL SCI | 7 | 0.4% |
| AMERICAN JOURNAL OF PHYSIOLOGY-LUNG CELLULAR AND MOLECULAR PHYSIOLOGY | AM J PHYSIOL-LUNG C | 6 | 0.3% |
| BMC PULMONARY MEDICINE | BMC PULM MED | 6 | 0.3% |
| CELLS | CELLS-BASEL | 6 | 0.3% |
| JOURNAL OF SURGICAL RESEARCH | J SURG RES | 6 | 0.3% |
| TRANSPLANT INTERNATIONAL | TRANSPLANT INT | 6 | 0.3% |
| CURRENT OPINION IN PULMONARY MEDICINE | CURR OPIN PULM MED | 5 | 0.3% |
| EXPERIMENTAL AND MOLECULAR PATHOLOGY | EXP MOL PATHOL | 5 | 0.3% |
| FRONTIERS IN MEDICINE | FRONT MED-LAUSANNE | 5 | 0.3% |
| GENERAL THORACIC AND CARDIOVASCULAR SURGERY | GEN THORAC CARDIOVAS | 5 | 0.3% |
| JOURNAL OF THORACIC IMAGING | J THORAC IMAG | 5 | 0.3% |
| RESPIROLOGY | RESPIROLOGY | 5 | 0.3% |
| ANNALS OF THE AMERICAN THORACIC SOCIETY | ANN AM THORAC SOC | 4 | 0.2% |
| ANNALS OF TRANSLATIONAL MEDICINE | ANN TRANSL MED | 4 | 0.2% |
| ANNALS OF TRANSPLANTATION | ANN TRANSPL | 4 | 0.2% |
| CLINICAL INFECTIOUS DISEASES | CLIN INFECT DIS | 4 | 0.2% |
| EUROPEAN JOURNAL OF RADIOLOGY | EUR J RADIOL | 4 | 0.2% |
| EUROPEAN RADIOLOGY | EUR RADIOL | 4 | 0.2% |
| EXPERIMENTAL AND CLINICAL TRANSPLANTATION | EXP CLIN TRANSPLANT | 4 | 0.2% |
| MODERN PATHOLOGY | MODERN PATHOL | 4 | 0.2% |
| RESPIRATION | RESPIRATION | 4 | 0.2% |
| SARCOIDOSIS VASCULITIS AND DIFFUSE LUNG DISEASES | SARCOIDOSIS VASC DIF | 4 | 0.2% |
| SWISS MEDICAL WEEKLY | SWISS MED WKLY | 4 | 0.2% |
| ANNALS OF SURGERY | ANN SURG | 3 | 0.2% |
| BMJ OPEN RESPIRATORY RESEARCH | BMJ OPEN RESPIR RES | 3 | 0.2% |
| CELLULAR IMMUNOLOGY | CELL IMMUNOL | 3 | 0.2% |
| CLINICAL AND TRANSLATIONAL GASTROENTEROLOGY | CLIN TRANSL GASTROEN | 3 | 0.2% |
| CURRENT CHALLENGES IN THORACIC SURGERY | CURR CHALL THORACIC | 3 | 0.2% |
| EUROPEAN RESPIRATORY REVIEW | EUR RESPIR REV | 3 | 0.2% |
| HUMAN PATHOLOGY | HUM PATHOL | 3 | 0.2% |
| JOURNAL OF CARDIOTHORACIC SURGERY | J CARDIOTHORAC SURG | 3 | 0.2% |
| JOURNAL OF CLINICAL GASTROENTEROLOGY | J CLIN GASTROENTEROL | 3 | 0.2% |
| JOURNAL OF CYSTIC FIBROSIS | J CYST FIBROS | 3 | 0.2% |
| JOURNAL OF EXPERIMENTAL MEDICINE | J EXP MED | 3 | 0.2% |
| LANCET | LANCET | 3 | 0.2% |
| PEDIATRIC PULMONOLOGY | PEDIATR PULM | 3 | 0.2% |
| PULMONARY PHARMACOLOGY & THERAPEUTICS | PULM PHARMACOL THER | 3 | 0.2% |
| RADIOLOGY-CARDIOTHORACIC IMAGING | RADIOL-CARDIOTHORAC | 3 | 0.2% |
| REVUE DES MALADIES RESPIRATOIRES | REV MAL RESPIR | 3 | 0.2% |
| SCANDINAVIAN CARDIOVASCULAR JOURNAL | SCAND CARDIOVASC J | 3 | 0.2% |
| STEM CELLS TRANSLATIONAL MEDICINE | STEM CELL TRANSL MED | 3 | 0.2% |
| SURGICAL ENDOSCOPY AND OTHER INTERVENTIONAL TECHNIQUES | SURG ENDOSC | 3 | 0.2% |
| TRANSPLANTATION REVIEWS | TRANSPLANT REV-ORLAN | 3 | 0.2% |
| AMERICAN JOURNAL OF ROENTGENOLOGY | AM J ROENTGENOL | 2 | 0.1% |
| AMERICAN JOURNAL OF TRANSLATIONAL RESEARCH | AM J TRANSL RES | 2 | 0.1% |
| ANNALS OF THORACIC AND CARDIOVASCULAR SURGERY | ANN THORAC CARDIOVAS | 2 | 0.1% |
| ARCHIVES OF PATHOLOGY & LABORATORY MEDICINE | ARCH PATHOL LAB MED | 2 | 0.1% |
| ARCHIVOS DE BRONCONEUMOLOGIA | ARCH BRONCONEUMOL | 2 | 0.1% |
| BIOCHEMICAL AND BIOPHYSICAL RESEARCH COMMUNICATIONS | BIOCHEM BIOPH RES CO | 2 | 0.1% |
| BMC INFECTIOUS DISEASES | BMC INFECT DIS | 2 | 0.1% |
| CHINESE MEDICAL JOURNAL | CHINESE MED J-PEKING | 2 | 0.1% |
| CLINICAL SCIENCE | CLIN SCI | 2 | 0.1% |
| CLINICS IN CHEST MEDICINE | CLIN CHEST MED | 2 | 0.1% |
| CURRENT MOLECULAR MEDICINE | CURR MOL MED | 2 | 0.1% |
| DISEASES OF THE ESOPHAGUS | DIS ESOPHAGUS | 2 | 0.1% |
| DRUGS | DRUGS | 2 | 0.1% |
| EBIOMEDICINE | EBIOMEDICINE | 2 | 0.1% |
| EUROPEAN JOURNAL OF CLINICAL PHARMACOLOGY | EUR J CLIN PHARMACOL | 2 | 0.1% |
| EXPERT REVIEW OF RESPIRATORY MEDICINE | EXPERT REV RESP MED | 2 | 0.1% |
| HISTOPATHOLOGY | HISTOPATHOLOGY | 2 | 0.1% |
| HLA | HLA | 2 | 0.1% |
| IMMUNOLOGIC RESEARCH | IMMUNOL RES | 2 | 0.1% |
| IMMUNOLOGY | IMMUNOLOGY | 2 | 0.1% |
| INSIGHTS INTO IMAGING | INSIGHTS IMAGING | 2 | 0.1% |
| INTERDISCIPLINARY CARDIOVASCULAR AND THORACIC SURGERY | INTERDISC CARDIOV TH | 2 | 0.1% |
| INTERNATIONAL JOURNAL OF CLINICAL AND EXPERIMENTAL PATHOLOGY | INT J CLIN EXP PATHO | 2 | 0.1% |
| JORNAL BRASILEIRO DE PNEUMOLOGIA | J BRAS PNEUMOL | 2 | 0.1% |
| JOURNAL OF BREATH RESEARCH | J BREATH RES | 2 | 0.1% |
| JOURNAL OF CLINICAL VIROLOGY | J CLIN VIROL | 2 | 0.1% |
| JOURNAL OF HEART TRANSPLANTATION | J HEART TRANSPLANT | 2 | 0.1% |
| JOURNAL OF PATHOLOGY | J PATHOL | 2 | 0.1% |
| JOURNAL OF THE AMERICAN COLLEGE OF SURGEONS | J AM COLL SURGEONS | 2 | 0.1% |
| LANCET RESPIRATORY MEDICINE | LANCET RESP MED | 2 | 0.1% |
| LIFE-BASEL | LIFE-BASEL | 2 | 0.1% |
| LUNG TRANSPLANTATION | EUR RESPIR MONOGR | 2 | 0.1% |
| MEDICINA CLINICA | MED CLIN-BARCELONA | 2 | 0.1% |
| MEDICINE | MEDICINE | 2 | 0.1% |
| MICROORGANISMS | MICROORGANISMS | 2 | 0.1% |
| NEUROGASTROENTEROLOGY AND MOTILITY | NEUROGASTROENT MOTIL | 2 | 0.1% |
| PROTEOMICS | PROTEOMICS | 2 | 0.1% |
| RESPIRATORY INVESTIGATION | RESPIR INVESTIG | 2 | 0.1% |
| SEMINARS IN IMMUNOPATHOLOGY | SEMIN IMMUNOPATHOL | 2 | 0.1% |
| THORACIC AND CARDIOVASCULAR SURGEON | THORAC CARDIOV SURG | 2 | 0.1% |
| THORACIC SURGERY CLINICS | THORAC SURG CLIN | 2 | 0.1% |
| TRANSFUSION | TRANSFUSION | 2 | 0.1% |
| VIRUSES-BASEL | VIRUSES-BASEL | 2 | 0.1% |
| ACADEMIC RADIOLOGY | ACAD RADIOL | 1 | 0.1% |
| ACS OMEGA | ACS OMEGA | 1 | 0.1% |
| ACTA ANAESTHESIOLOGICA SCANDINAVICA | ACTA ANAESTH SCAND | 1 | 0.1% |
| ACTA CLINICA BELGICA | ACTA CLIN BELG | 1 | 0.1% |
| ADVANCES IN RESPIRATORY MEDICINE | ADV RESPIR MED | 1 | 0.1% |
| ADVANCES IN THERAPY | ADV THER | 1 | 0.1% |
| AGING-US | AGING-US | 1 | 0.1% |
| AMERICAN JOURNAL OF HEALTH-SYSTEM PHARMACY | AM J HEALTH-SYST PH | 1 | 0.1% |
| AMERICAN JOURNAL OF MEDICINE | AM J MED | 1 | 0.1% |
| AMERICAN JOURNAL OF SURGICAL PATHOLOGY | AM J SURG PATHOL | 1 | 0.1% |
| AMERICAN JOURNAL OF THE MEDICAL SCIENCES | AM J MED SCI | 1 | 0.1% |
| AMERICAN REVIEW OF RESPIRATORY DISEASE | AM REV RESPIR DIS | 1 | 0.1% |
| AMERICAN SURGEON | AM SURGEON | 1 | 0.1% |
| ANGIOLOGY | ANGIOLOGY | 1 | 0.1% |
| ANNALS OF INTERNAL MEDICINE | ANN INTERN MED | 1 | 0.1% |
| ANNALS OF MEDICINE | ANN MED | 1 | 0.1% |
| ANNALS OF NUCLEAR MEDICINE | ANN NUCL MED | 1 | 0.1% |
| ANNALS OF SAUDI MEDICINE | ANN SAUDI MED | 1 | 0.1% |
| ANTIVIRAL THERAPY | ANTIVIR THER | 1 | 0.1% |
| ARCHIVES OF DISEASE IN CHILDHOOD | ARCH DIS CHILD | 1 | 0.1% |
| ARCHIVES OF MEDICAL RESEARCH | ARCH MED RES | 1 | 0.1% |
| ARCHIVES OF SURGERY | ARCH SURG-CHICAGO | 1 | 0.1% |
| AUSTRALIAN AND NEW ZEALAND JOURNAL OF MEDICINE | AUST NZ J MED | 1 | 0.1% |
| BIODRUGS | BIODRUGS | 1 | 0.1% |
| BIOENGINEERING-BASEL | BIOENGINEERING-BASEL | 1 | 0.1% |
| BIOMARKERS | BIOMARKERS | 1 | 0.1% |
| BIOMARKERS IN MEDICINE | BIOMARK MED | 1 | 0.1% |
| BIOMEDICINES | BIOMEDICINES | 1 | 0.1% |
| BIOMETRICAL JOURNAL | BIOMETRICAL J | 1 | 0.1% |
| BIOMETRICS | BIOMETRICS | 1 | 0.1% |
| BIOTECHNOLOGY LETTERS | BIOTECHNOL LETT | 1 | 0.1% |
| BMC MICROBIOLOGY | BMC MICROBIOL | 1 | 0.1% |
| CANADIAN RESPIRATORY JOURNAL | CAN RESPIR J | 1 | 0.1% |
| CARDIOVASCULAR PATHOLOGY | CARDIOVASC PATHOL | 1 | 0.1% |
| CELL AND TISSUE RESEARCH | CELL TISSUE RES | 1 | 0.1% |
| CELL REPORTS MEDICINE | CELL REP MED | 1 | 0.1% |
| CELLULAR AND MOLECULAR LIFE SCIENCES | CELL MOL LIFE SCI | 1 | 0.1% |
| CIRCULATION | CIRCULATION | 1 | 0.1% |
| CLINICAL & TRANSLATIONAL IMMUNOLOGY | CLIN TRANSL IMMUNOL | 1 | 0.1% |
| CLINICAL AND EXPERIMENTAL ALLERGY | CLIN EXP ALLERGY | 1 | 0.1% |
| CLINICAL AND EXPERIMENTAL PHARMACOLOGY AND PHYSIOLOGY | CLIN EXP PHARMACOL P | 1 | 0.1% |
| CLINICAL AND PRECLINICAL OPTICAL DIAGNOSTICS II | PROC SPIE | 1 | 0.1% |
| CLINICAL EPIGENETICS | CLIN EPIGENETICS | 1 | 0.1% |
| CLINICAL IMMUNOLOGY | CLIN IMMUNOL | 1 | 0.1% |
| CLINICAL MICROBIOLOGY AND INFECTION | CLIN MICROBIOL INFEC | 1 | 0.1% |
| CLINICAL NUCLEAR MEDICINE | CLIN NUCL MED | 1 | 0.1% |
| CLINICAL PHYSIOLOGY AND FUNCTIONAL IMAGING | CLIN PHYSIOL FUNCT I | 1 | 0.1% |
| CLINICAL RESPIRATORY JOURNAL | CLIN RESPIR J | 1 | 0.1% |
| COCHRANE DATABASE OF SYSTEMATIC REVIEWS | COCHRANE DB SYST REV | 1 | 0.1% |
| COMMUNICATIONS MEDICINE | COMMUN MED-LONDON | 1 | 0.1% |
| COMPLEMENT THERAPEUTICS | ADV EXP MED BIOL | 1 | 0.1% |
| COMPUTERS IN BIOLOGY AND MEDICINE | COMPUT BIOL MED | 1 | 0.1% |
| COPD-JOURNAL OF CHRONIC OBSTRUCTIVE PULMONARY DISEASE | COPD | 1 | 0.1% |
| CRITICAL REVIEWS IN IMMUNOLOGY | CRIT REV IMMUNOL | 1 | 0.1% |
| CUREUS JOURNAL OF MEDICAL SCIENCE | CUREUS J MED SCIENCE | 1 | 0.1% |
| CURRENT INFECTIOUS DISEASE REPORTS | CURR INFECT DIS REP | 1 | 0.1% |
| CURRENT MEDICINAL CHEMISTRY | CURR MED CHEM | 1 | 0.1% |
| CURRENT PHARMACEUTICAL DESIGN | CURR PHARM DESIGN | 1 | 0.1% |
| CURRENT PULMONOLOGY REPORTS | CURR PULMONOL REP | 1 | 0.1% |
| CURRENT TRANSPLANTATION REPORTS | CURR TRANSPLANT REP | 1 | 0.1% |
| CYTOKINE | CYTOKINE | 1 | 0.1% |
| DIAGNOSTICS | DIAGNOSTICS | 1 | 0.1% |
| DISEASE MARKERS | DIS MARKERS | 1 | 0.1% |
| DISEASE MARKERS IN EXHALED BREATH: BASIC MECHANISMS AND CLINICAL APPLICATIONS | NATO SCI SER I LIFE | 1 | 0.1% |
| ECLINICALMEDICINE | ECLINICALMEDICINE | 1 | 0.1% |
| EUROPEAN JOURNAL OF HUMAN GENETICS | EUR J HUM GENET | 1 | 0.1% |
| EUROPEAN JOURNAL OF IMMUNOLOGY | EUR J IMMUNOL | 1 | 0.1% |
| EUROPEAN JOURNAL OF PEDIATRIC SURGERY | EUR J PEDIATR SURG | 1 | 0.1% |
| EUROPEAN JOURNAL OF PHARMACEUTICS AND BIOPHARMACEUTICS | EUR J PHARM BIOPHARM | 1 | 0.1% |
| EUROPEAN JOURNAL OF PHARMACOLOGY | EUR J PHARMACOL | 1 | 0.1% |
| EXPERT OPINION ON DRUG METABOLISM & TOXICOLOGY | EXPERT OPIN DRUG MET | 1 | 0.1% |
| EXPERT OPINION ON PHARMACOTHERAPY | EXPERT OPIN PHARMACO | 1 | 0.1% |
| EXPERT REVIEW OF MOLECULAR DIAGNOSTICS | EXPERT REV MOL DIAGN | 1 | 0.1% |
| FASEB JOURNAL | FASEB J | 1 | 0.1% |
| FRONTIERS IN PHYSIOLOGY | FRONT PHYSIOL | 1 | 0.1% |
| FUNDAMENTAL & CLINICAL PHARMACOLOGY | FUND CLIN PHARMACOL | 1 | 0.1% |
| GASTROENTEROLOGY RESEARCH | GASTROENTEROL RES | 1 | 0.1% |
| GENETICS AND MOLECULAR RESEARCH | GENET MOL RES | 1 | 0.1% |
| HEART SURGERY FORUM | HEART SURG FORUM | 1 | 0.1% |
| HELIYON | HELIYON | 1 | 0.1% |
| HISTOLOGY AND HISTOPATHOLOGY | HISTOL HISTOPATHOL | 1 | 0.1% |
| HUMAN GENE THERAPY | HUM GENE THER | 1 | 0.1% |
| IEEE TRANSACTIONS ON BIOMEDICAL ENGINEERING | IEEE T BIO-MED ENG | 1 | 0.1% |
| IMMUNITY INFLAMMATION AND DISEASE | IMMUN INFLAMM DIS | 1 | 0.1% |
| INDIAN JOURNAL OF THORACIC AND CARDIOVASCULAR SURGERY | INDIAN J THORAC CARD | 1 | 0.1% |
| INFECTIOUS DISEASES AND THERAPY | INFECT DIS THER | 1 | 0.1% |
| INFLAMMATION RESEARCH | INFLAMM RES | 1 | 0.1% |
| INTERNAL MEDICINE JOURNAL | INTERN MED J | 1 | 0.1% |
| INTERNATIONAL ARCHIVES OF ALLERGY AND IMMUNOLOGY | INT ARCH ALLERGY IMM | 1 | 0.1% |
| INTERNATIONAL JOURNAL OF CLINICAL AND EXPERIMENTAL MEDICINE | INT J CLIN EXP MED | 1 | 0.1% |
| INTERNATIONAL JOURNAL OF RADIATION ONCOLOGY BIOLOGY PHYSICS | INT J RADIAT ONCOL | 1 | 0.1% |
| ISRAEL MEDICAL ASSOCIATION JOURNAL | ISRAEL MED ASSOC J | 1 | 0.1% |
| JAMA SURGERY | JAMA SURG | 1 | 0.1% |
| JOURNAL OF ALLERGY AND CLINICAL IMMUNOLOGY | J ALLERGY CLIN IMMUN | 1 | 0.1% |
| JOURNAL OF ARTIFICIAL ORGANS | J ARTIF ORGANS | 1 | 0.1% |
| JOURNAL OF BIOMEDICAL OPTICS | J BIOMED OPT | 1 | 0.1% |
| JOURNAL OF CARDIOPULMONARY REHABILITATION AND PREVENTION | J CARDIOPULM REHABIL | 1 | 0.1% |
| JOURNAL OF CARDIOVASCULAR PHARMACOLOGY | J CARDIOVASC PHARM | 1 | 0.1% |
| JOURNAL OF CLINICAL APHERESIS | J CLIN APHERESIS | 1 | 0.1% |
| JOURNAL OF CLINICAL PATHOLOGY | J CLIN PATHOL | 1 | 0.1% |
| JOURNAL OF COMPUTER ASSISTED TOMOGRAPHY | J COMPUT ASSIST TOMO | 1 | 0.1% |
| JOURNAL OF FUNGI | J FUNGI | 1 | 0.1% |
| JOURNAL OF GASTROINTESTINAL SURGERY | J GASTROINTEST SURG | 1 | 0.1% |
| JOURNAL OF IMMUNOLOGY RESEARCH | J IMMUNOL RES | 1 | 0.1% |
| JOURNAL OF INFECTIOUS DISEASES | J INFECT DIS | 1 | 0.1% |
| JOURNAL OF INFLAMMATION RESEARCH | J INFLAMM RES | 1 | 0.1% |
| JOURNAL OF INVESTIGATIVE MEDICINE | J INVEST MED | 1 | 0.1% |
| JOURNAL OF LABORATORY AND CLINICAL MEDICINE | J LAB CLIN MED | 1 | 0.1% |
| JOURNAL OF MAGNETIC RESONANCE IMAGING | J MAGN RESON IMAGING | 1 | 0.1% |
| JOURNAL OF MASS SPECTROMETRY | J MASS SPECTROM | 1 | 0.1% |
| JOURNAL OF MEDICAL ECONOMICS | J MED ECON | 1 | 0.1% |
| JOURNAL OF PATHOLOGY CLINICAL RESEARCH | J PATHOL CLIN RES | 1 | 0.1% |
| JOURNAL OF PHARMACY PRACTICE | J PHARM PRACT | 1 | 0.1% |
| JOURNAL OF PROTEOME RESEARCH | J PROTEOME RES | 1 | 0.1% |
| JOURNAL OF SCLERODERMA AND RELATED DISORDERS | J SCLERODERMA RELAT | 1 | 0.1% |
| JOURNAL OF SURGICAL CASE REPORTS | J SURG CASE REP | 1 | 0.1% |
| JOURNAL OF TRANSLATIONAL MEDICINE | J TRANSL MED | 1 | 0.1% |
| JOVE-JOURNAL OF VISUALIZED EXPERIMENTS | JOVE-J VIS EXP | 1 | 0.1% |
| JTCVS OPEN | JTCVS OPEN | 1 | 0.1% |
| KARDIOCHIRURGIA I TORAKOCHIRURGIA POLSKA-POLISH JOURNAL OF THORACIC AND CARDIOVASCULAR SURGERY | KARDIOCHIR TORAKOCHI | 1 | 0.1% |
| LEUKEMIA & LYMPHOMA | LEUKEMIA LYMPHOMA | 1 | 0.1% |
| LIFE SCIENCES | LIFE SCI | 1 | 0.1% |
| MACROLIDES AS IMMUNOMODULATORY AGENTS | PROG INFLAMM RES SER | 1 | 0.1% |
| MATHEMATICAL AND COMPUTER MODELLING | MATH COMPUT MODEL | 1 | 0.1% |
| MAYO CLINIC PROCEEDINGS | MAYO CLIN PROC | 1 | 0.1% |
| MEDIATORS OF INFLAMMATION | MEDIAT INFLAMM | 1 | 0.1% |
| MEDICAL JOURNAL OF AUSTRALIA | MED J AUSTRALIA | 1 | 0.1% |
| MICROBIOME | MICROBIOME | 1 | 0.1% |
| MICROSURGERY | MICROSURG | 1 | 0.1% |
| MONATSSCHRIFT KINDERHEILKUNDE | MONATSSCHR KINDERH | 1 | 0.1% |
| MSYSTEMS | MSYSTEMS | 1 | 0.1% |
| MUCOSAL IMMUNOLOGY | MUCOSAL IMMUNOL | 1 | 0.1% |
| NANOMEDICINE | NANOMEDICINE-UK | 1 | 0.1% |
| NANOTOXICOLOGY | NANOTOXICOLOGY | 1 | 0.1% |
| NEW ENGLAND JOURNAL OF MEDICINE | NEW ENGL J MED | 1 | 0.1% |
| NUCLEAR MEDICINE COMMUNICATIONS | NUCL MED COMMUN | 1 | 0.1% |
| PANMINERVA MEDICA | PANMINERVA MED | 1 | 0.1% |
| PATHOLOGE | PATHOLOGE | 1 | 0.1% |
| PEDIATRIC DRUGS | PEDIATR DRUGS | 1 | 0.1% |
| PHARMACOGENOMICS JOURNAL | PHARMACOGENOMICS J | 1 | 0.1% |
| PHARMACOLOGICAL REVIEWS | PHARMACOL REV | 1 | 0.1% |
| PHARMACOLOGY & THERAPEUTICS | PHARMACOL THERAPEUT | 1 | 0.1% |
| PRESSE MEDICALE | PRESSE MED | 1 | 0.1% |
| PROCEEDINGS OF THE 19TH ANNUAL INTERNATIONAL CONFERENCE OF THE IEEE ENGINEERING IN MEDICINE AND BIOLOGY SOCIETY, VOL 19, PTS 1-6: MAGNIFICENT MILESTONES AND EMERGING OPPORTUNITIES IN MEDICAL ENGINEERING | P ANN INT IEEE EMBS | 1 | 0.1% |
| PROCEEDINGS OF THE NATIONAL ACADEMY OF SCIENCES OF THE UNITED STATES OF AMERICA | P NATL ACAD SCI USA | 1 | 0.1% |
| PROGRESS IN TRANSPLANTATION | PROG TRANSPLANT | 1 | 0.1% |
| PSYCHOSOMATIC MEDICINE | PSYCHOSOM MED | 1 | 0.1% |
| PULMONARY CIRCULATION | PULM CIRC | 1 | 0.1% |
| RADIOLOGICAL PHYSICS AND TECHNOLOGY | RADIOL PHYS TECHNOL | 1 | 0.1% |
| RESPIRATORY MEDICINE AND RESEARCH | RESPIR MED RES-FRANC | 1 | 0.1% |
| RESPIRATORY PHYSIOLOGY & NEUROBIOLOGY | RESP PHYSIOL NEUROBI | 1 | 0.1% |
| RESPIROLOGY CASE REPORTS | RESPIROL CASE REP | 1 | 0.1% |
| SCIENCE ADVANCES | SCI ADV | 1 | 0.1% |
| SEMINARS IN IMMUNOLOGY | SEMIN IMMUNOL | 1 | 0.1% |
| STEM CELLS INTERNATIONAL | STEM CELLS INT | 1 | 0.1% |
| SURGERY | SURGERY | 1 | 0.1% |
| THERAPEUTIC ADVANCES IN CHRONIC DISEASE | THER ADV CHRONIC DIS | 1 | 0.1% |
| THERAPEUTIC ADVANCES IN RESPIRATORY DISEASE | THER ADV RESPIR DIS | 1 | 0.1% |
| TISSUE ANTIGENS | TISSUE ANTIGENS | 1 | 0.1% |
| TRANSFUSION MEDICINE | TRANSFUSION MED | 1 | 0.1% |
| TRANSFUSIONSMEDIZIN | TRANSFUSIONSMEDIZIN | 1 | 0.1% |
| TRANSLATIONAL RESEARCH | TRANSL RES | 1 | 0.1% |
| ULTRASTRUCTURAL PATHOLOGY | ULTRASTRUCT PATHOL | 1 | 0.1% |
| VASCULAR SURGERY | VASCULAR SURG | 1 | 0.1% |
| VIDEOSURGERY AND OTHER MINIINVASIVE TECHNIQUES | VIDEOSURGERY MINIINV | 1 | 0.1% |
| WORLD JOURNAL OF PEDIATRICS | WORLD J PEDIATR | 1 | 0.1% |
| YONSEI MEDICAL JOURNAL | YONSEI MED J | 1 | 0.1% |
| ZEITSCHRIFT FUR HERZ THORAX UND GEFASSCHIRURGIE | Z HERZ THORAX GEFASS | 1 | 0.1% |
| ZENTRALBLATT FUR CHIRURGIE | ZBL CHIR | 1 | 0.1% |

*† n: number of papers from that journal. % corpus: proportion of the full corpus. Journals sorted by descending publication count. Language distribution: of the 1,851 corpus papers, 1,841 (99.5%) were in English; the remaining 10 non-English papers comprised 4 in German, 3 in French, and 3 in Spanish.*

**Supplementary Table 2. Mapping of the 24 BERTopic thematic clusters to four research domains.**

| **Research domain** | **Topic** | **n** | **% corpus** |
| --- | --- | --- | --- |
| Basic/Translational | Preclinical models of obliterative bronchiolitis | 262 | 14.15% |
|  | Translational studies on CLAD pathogenesis | 145 | 7.83% |
|  | Obliterative bronchiolitis — pathophysiology and overview | 88 | 4.75% |
|  | Genetic polymorphisms and HLA-G | 42 | 2.27% |
|  | Extracellular vesicles and self-antigens | 20 | 1.08% |
| Diagnostics/Monitoring | Donor-specific antibodies and antibody-mediated rejection | 125 | 6.75% |
|  | Peripheral blood immune profiling in BOS | 106 | 5.73% |
|  | BAL neutrophilia and NRAD | 74 | 4% |
|  | Respiratory viral infections and CLAD risk | 54 | 2.92% |
|  | CT imaging — air trapping and BOS diagnosis | 41 | 2.22% |
|  | Airway microbiome and bacterial colonization | 41 | 2.22% |
|  | Surveillance bronchoscopy and transbronchial biopsy | 40 | 2.16% |
|  | Donor-derived cell-free DNA as biomarker | 22 | 1.19% |
| Therapeutics | Calcineurin inhibitor and mTOR immunosuppression | 98 | 5.29% |
|  | Gastroesophageal reflux and aspiration | 68 | 3.67% |
|  | Azithromycin treatment | 43 | 2.32% |
|  | Induction immunosuppression | 36 | 1.94% |
|  | Extracorporeal photopheresis (ECP) | 31 | 1.67% |
|  | CMV infection and antiviral prophylaxis | 24 | 1.3% |
| Clinical/Outcomes | Retransplantation and long-term survival | 165 | 8.91% |
|  | RAS / restrictive phenotype and CLAD classification | 128 | 6.92% |
|  | BOS staging and FEV1 decline | 121 | 6.54% |
|  | CLAD management — general reviews and treatment options | 53 | 2.86% |
|  | Cystic fibrosis as transplant indication | 24 | 1.3% |

*Domains assigned by author consensus. n: papers assigned to the topic; % corpus of the full corpus (n 1,851).*

*Domain scope: basic/translational (disease mechanism, preclinical models, genetics); diagnostics/monitoring (biomarkers, imaging, surveillance, immune profiling); therapeutics (drug regimens, procedures, prophylaxis); clinical/outcomes (phenotype classification, survival, transplant indications).*

**Supplementary Table 3. Geographic distribution of the CLAD literature.**

**(A) Publications by continent**

| **Continent** | **n papers** | **% of papers** |
| --- | --- | --- |
| North America | 922 | 50.7% |
| Europe | 779 | 42.8% |
| Asia | 210 | 11.5% |
| Oceania | 144 | 7.9% |
| South America | 11 | 0.6% |
| Africa | 3 | 0.2% |

**(B) Top 20 publishing cities**

| **Rank** | **City** | **Country** | **Continent** | **n papers** | **% of papers** |
| --- | --- | --- | --- | --- | --- |
| 1 | Leuven | Belgium | Europe | 141 | 7.8% |
| 2 | St Louis | United States | North America | 139 | 7.6% |
| 3 | Toronto | Canada | North America | 131 | 7.2% |
| 4 | Durham | United States | North America | 100 | 5.5% |
| 5 | Los Angeles | United States | North America | 80 | 4.4% |
| 6 | Hannover | Germany | Europe | 79 | 4.3% |
| 7 | Pittsburgh | United States | North America | 69 | 3.8% |
| 8 | Stanford | United States | North America | 64 | 3.5% |
| 9 | Baltimore | United States | North America | 61 | 3.4% |
| 10 | Boston | United States | North America | 61 | 3.4% |
| 11 | Melbourne | Australia | Oceania | 61 | 3.4% |
| 12 | Paris | France | Europe | 61 | 3.4% |
| 13 | Philadelphia | United States | North America | 55 | 3% |
| 14 | Newcastle upon Tyne | United Kingdom | Europe | 49 | 2.7% |
| 15 | Phoenix | United States | North America | 49 | 2.7% |
| 16 | Vienna | Austria | Europe | 47 | 2.6% |
| 17 | Zurich | Switzerland | Europe | 44 | 2.4% |
| 18 | New York | United States | North America | 43 | 2.4% |
| 19 | Minneapolis | United States | North America | 42 | 2.3% |
| 20 | San Francisco | United States | North America | 42 | 2.3% |

*Continent and city parsed from Web of Science author-affiliation addresses. Greater-Paris (Île-de-France) institutions were aggregated to “Paris”. Each paper is attributed to every continent and city represented among its affiliations; shares therefore sum to >100% because internationally co-authored papers contribute to more than one continent/city. Denominator: 1,819 of 1,851 papers with parseable addresses.*
